# Protective Effects of Cannabidivarin and Cannabigerol on Cells of the Blood–Brain Barrier Under Ischemic Conditions

**DOI:** 10.1089/can.2020.0159

**Published:** 2021-08-05

**Authors:** Nicole L. Stone, Timothy J. England, Saoirse E. O'Sullivan

**Affiliations:** ^1^ Division of Medical Sciences and Graduate Entry Medicine, School of Medicine, University of Nottingham, Royal Derby Hospital, Derby, United Kingdom.; ^2^ University Hospitals of Derby and Burton NHS Foundation Trust, Royal Derby Hospital, Derby, United Kingdom.; ^3^ CanPharmaConsulting, Nottingham, United Kingdom.

**Keywords:** blood–brain barrier, cannabidivarin, cannabigerol, cannabinoids, ischemia, neuroprotection

## Abstract

**Background and Objectives:** Preclinical studies have shown cannabidiol is protective in models of ischemic stroke. Based on results from our recent systematic review, we investigated the effects of two promising neuroprotective phytocannabinoids, cannabigerol (CBG) and cannabidivarin (CBDV), on cells of the blood–brain barrier (BBB), namely human brain microvascular endothelial cells (HBMECs), pericytes, and astrocytes.

**Experimental Approach:** Cultures were subjected to oxygen-glucose deprivation (OGD) protocol to model ischemic stroke and cell culture medium was assessed for cytokines and adhesion molecules post-OGD. Astrocyte cell lysates were also analyzed for DNA damage markers. Antagonist studies were conducted where appropriate to study receptor mechanisms.

**Results:** In astrocytes CBG and CBDV attenuated levels of interleukin-6 (IL-6) and lactate dehydrogenase (LDH), whereas CBDV (10 nM–10 μM) also decreased vascular endothelial growth factor (VEGF) secretion. CBDV (300 nM–10 μM) attenuated levels of monocyte chemoattractant protein (MCP)-1 in HBMECs. In astrocytes, CBG decreased levels of DNA damage proteins, including p53, whereas CBDV increased levels of DNA damage markers. Antagonists for CB_1_, CB_2_, PPAR-γ, PPAR-α, 5-HT1_A_, and TRPV1 had no effect on CBG (3 μM) or CBDV (1 μM)-mediated decreases in LDH in astrocytes. GPR55 and GPR18 were partially implicated in the effects of CBDV, but no molecular target was identified for CBG.

**Conclusions:** We show that CBG and CBDV were protective against OG mediated injury in three different cells that constitute the BBB, modulating different hallmarks of ischemic stroke pathophysiology. These data enhance our understanding of the protective effects of CBG and CBDV and warrant further investigation into these compounds in ischemic stroke. Future studies should identify other possible neuroprotective effects of CBG and CBDV and their corresponding mechanisms of action.

## Introduction

The blood–brain barrier (BBB) is a unique interface that separates the central nervous system (CNS) and the periphery, protecting the brain from damaging components found in general circulation, namely peripheral leukocytes, macromolecules, and xenobiotics.^1,2^ The barrier itself is formed by microvascular endothelial cells, which are encompassed by pericytes, and altogether surrounded by astrocyte end feet, which cover 99% of BBB endothelia.^3^ Cerebral ischemia–reperfusion (IR) initiates a plethora of inflammatory signaling pathways, cytotoxic glutamate release, and oxidative stress, all of which contribute to increases in BBB permeability.^4^ This loss of BBB integrity ultimately causes uncontrolled immune infiltration into the CNS that perpetuates neuronal injury and hinders poststroke recovery. Although administration of tissue plasminogen activator (tPA) and mechanical thrombectomy are effective licensed therapies to dissolve or remove the culpable clot, at present, there are no available approved therapies that mitigate poststroke injury.^5^

Cannabidiol (CBD), one of the chemicals found in *Cannabis sativa*, has displayed a range of neuroprotective qualities, preventing neuronal loss,^6,7^ attenuating astrocyte reactivity,^8^ and dampening the neuroinflammatory response.^9^ Unlike delta^9^-tetrahydrocannabinol (Δ^9^-THC), CBD does not activate the central cannabinoid receptors, CB_1_ or CB_2_, but activates a plethora of other targets including PPAR-γ, TRPV1, and 5-HT_1A_ receptors.^10–13^ CBD has formulations (alone and with Δ^9^-THC) licensed by GW pharmaceuticals to treat rare childhood epilepsies and spasticity associated with multiple sclerosis. The protective effects of CBD in stroke models has been well documented,^14^ specifically CBD has been shown to reduce infarct volume,^15,16^ reduce glutamate toxicity,^9,17^ attenuate mitochondrial dysfunction^18^ and glial activation.^6,19^ In a co-culture BBB model CBD preserved barrier integrity after oxygen-glucose deprivation (OGD), which was mediated at least in part by PPAR-γ and 5-HT_1A_ receptors.^12^

Cannabigerol (CBG) and cannabidivarin (CBDV) are neutral cannabinoids present in cannabis and studies have found these compounds share similar pharmacological characteristics to CBD. Like CBD, they do not produce feelings of euphoria and display antioxidant and anti-inflammatory properties, as well as interacting with a range of target proteins including TRPV1,^13^ PPAR-γ,^20^ 5-HT_1A_, and CB_2_.^21^ Recently our group conducted a systematic review focusing on the neuroprotective properties of minor phytocannabinoids (other than Δ^9^-THC or CBD) and found that CBG and CBDV show efficacy in models of Huntington's disease, Alzheimer's, and epilepsy, with CBG mediating its protective effects through PPAR-γ activation,^22^ the same mechanism by which we have shown that CBD protects BBB integrity.^12^ However, despite these compounds having neuroprotective effects in other models, no studies have been conducted to test whether CBG or CBDV are protective in IR injury.

In light of the above, we hypothesized these compounds may exhibit protective properties at the BBB in a stroke model. To test this, we treated cells of the BBB with CBG or CBDV *in vitro* before an OGD protocol and measured various proinflammatory cytokines, adhesion molecules, and cell damage markers.

## Materials and Methods

### Materials

CBG and CBDV were kindly gifted by STI pharmaceuticals. Both compounds were dissolved in 100% ethanol to 10 mM and were stored at −20°C. AM251, AM630, GW6471, GW9962, O1918, CID16020046, SB366791 (Tocris, United Kingdom) were dissolved in dimethyl sulfoxide as stock solutions of 10 mM. (S)-WAY100135 was dissolved in deionized water. Antagonists were stored at −20°C and dilutions were made fresh as required.

### General cell culture

Human brain microvascular endothelial cells (HBMECs), astrocytes, and pericytes (passages 3–6) were grown in their respective medium and maintained at 37°C in a humidified incubator supplemented with 5% CO_2_. HBMECs were cultured on fibronectin-coated plasticware (2 μg/cm^2^), as per supplier recommendations. Primary cells and medium were purchased from ScienCell, United Kingdom.

### OGD protocol

To simulate ischemic conditions, normal medium was replaced with glucose free RPMI medium (Gibco, United Kingdom) containing either CBG or CBDV (10 nM to 10 μM), alongside a vehicle control (0.01% ethanol). Cell culture plates were then placed in an anoxic bag (BD GasPak™, anaerobe) for 4 h (8 h for astrocyte experiments) plus an additional 20 min to ensure anaerobic conditions. For vehicle normoxia, ethanol (0.01%) was added to the respective medium of each cell type (ScienCell) and maintained in normal oxygenated conditions. After OGD, medium was aspirated and replaced with each cell types respective medium (ScienCell) containing the relevant concentrations of CBG or CBDV for a 20-h/16-h reperfusion period. At 24 h, the medium was sampled, and cells were lysed with RIPA buffer containing protease and phosphatase inhibitors (Sigma, United Kingdom; ThermoFisher, United Kingdom). Medium and lysates were stored at −80°C for future analysis.

### Total protein

To quantify total protein, a bicinchoninic acid (BCA) protein assay was performed on cell lysates. A working reagent of copper II sulfate and BCA (Sigma-Aldrich) was prepared in a 1:50 ratio and added to wells. After a 30-min incubation at 37°C, plates were read at 562 nm. Unknowns were extrapolated from a standard curve of known concentrations of bovine serum album. Unless otherwise stated, all secreted and intracellular proteins were normalized to total protein.

### Enzyme-linked immunosorbent assay

Medium samples were analyzed for various proinflammatory cytokines including interleukin (IL)-6, IL-8, and adhesion molecules including intracellular adhesion molecule (ICAM)-1, vascular endothelial growth factor (VEGF), monocyte chemoattractant protein (MCP)-1 using duo-set enzyme-linked immunosorbent assay (ELISA) by R&D systems, United Kingdom (DY206, DY208, DY720, DY293B, and DY279). Raw values at 570 nm were subtracted from values obtained at 450 nm, sample concentrations were determined by extrapolating unknowns from the 8-point standard curve (known concentrations).

### Lactate dehydrogenase assay

A lactate dehydrogenase (LDH) assay was performed to determine nonspecific damage induced by the OGD protocol. A standard curve of known concentration of nicotinamide adenine dinucleotide was constructed as per manufacturer's instructions. Fifty microliters of standard or sample was aliquoted into a 96-well plate and 50 μL of assay mix was added. Plate absorbance was read at 450 nm and unknown values were obtained from a standard curve.

### DNA damage/genotoxicity assay

Astrocyte lysates post-OGD were analyzed using the Milliplex DNA damage/Genotoxicity multiplex assay kit (Millipore, 48–621MAG) to detect changes in DNA damage markers ataxia-telangiectasia mutated (ATR-Total), checkpoint kinases 1, 2 (Chk1, Ser345 and Chk2, and Thr68), histone family member X (H2A.X, Ser139), mouse double minute 2 homolog (MDM2, total), cyclin-dependent kinase inhibitor 1 (p21, total), tumour protein (p53, Ser15). Kits were performed according to manufacturer's instructions.

### Statistical analysis

All data are represented as the mean±standard error of the mean, data were assessed for normality using the D'Agostino–Pearson normality test and subsequently analyzed using one-way analysis of variance with Dunnett's *post hoc* analysis. All statistical analyses were conducted using GraphPad prism (7/8) (Version 7.01; GraphPad Software, Inc.), comparing either vehicle normoxia or vehicle OGD with all other treatments. A value of *p*<0.05 was considered significant.

## Results

### HBMEC monocultures

Protein levels from HBMEC lysates were significantly lower post-OGD compared with vehicle normoxia wells (*p*<0.001). This was not affected by pretreatment with CBDV or CBG (Supplementary Fig. S1C, F).

**FIG. 1.**
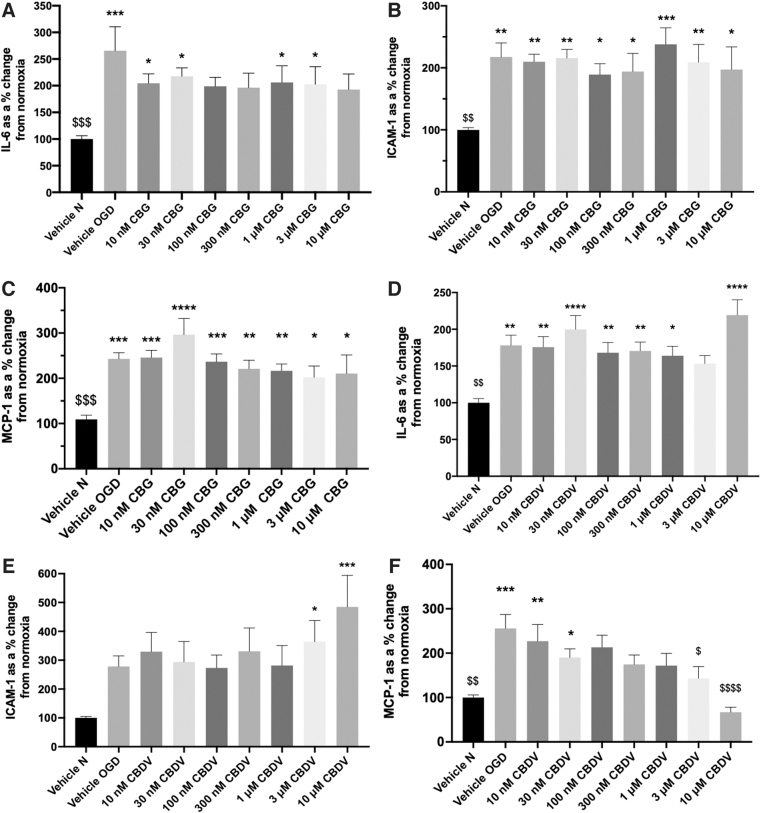
The effects of CBG and CBDV on HBMEC monocultures. Medium was analyzed for IL-6 **(A, D)**, ICAM-1 **(B, E),** and MCP-1 **(C, F)** 24 h after 4-h OGD. Data were normalized to total protein (calculated using a BCA assay) and are given as a % change from the normoxia vehicle presented as means with error bars representing SEM. *n*=6–9 from three experimental repeats. *, Significant difference compared with vehicle normoxia (vehicle N) (*p<0.05, **p<0.01, ***p<0.001, and ****p<0.0001). ^$^*p* < 0.05, ^$$^*p* < 0.01, ^$$$^*p* < 0.001, and ^$$$$^*p* < 0.0001) significant difference to vehicle OGD, one-way ANOVA with Dunnett's *post hoc* analysis. ANOVA, analysis of variance; BCA, bicinchoninic acid; CBDV, cannabidivarin; CBG, cannabigerol; HBMEC, human brain microvascular endothelial cell; ICAM-1, intracellular adhesion molecule-1; IL-6, interleukin-6; MCP-1, monocyte chemoattractant protein-1; OGD, oxygen-glucose deprivation; SEM, standard error of the mean.

IL-6, ICAM-1, and MCP-1 were significantly increased in cell culture medium 24 h after 4-h OGD compared with normoxia vehicle (*p*<0.05; Fig. 1A–F). Pretreatment with CBG (10 nM–10 μM) displayed an overall trend to decrease IL-6 and 100 nM, 300 nM, and 10 μM CBG-treated wells were not statistically significant to vehicle normoxia (Fig. 1A). CBG pretreatment did not alter ICAM-1 and MCP-1 secretion in response to OGD (Fig. 1B, C).

Pretreatment with CBDV (10 nM–1 μM and 10 μM) did not attenuate IL-6 levels 24-h post-OGD. However, 3 μM CBDV was not significantly different from vehicle normoxia (Figure 1D). Pretreatment with 3 and 10 μM CBDV significantly increased levels of ICAM-1 24-h post-OGD (*p*<0.05, Fig. 1E). CBDV (100 nM–10 μM) concentration-dependently reduced levels of MCP-1, an effect that was significantly different to vehicle OGD at 3 and 10 μM (*p*<0.05; Fig. 1F).

### Pericyte monocultures

**FIG. 2.**
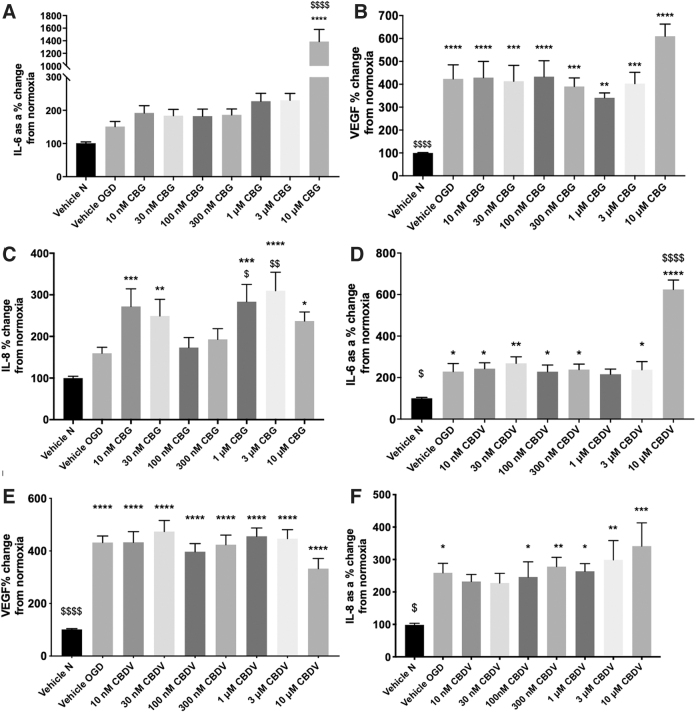
The effects of CBDV and CBG on pericyte monocultures. Medium 24 h after 4-h OGD was analyzed for IL-6, VEGF, and IL-8 **(A–F)**. Data were normalized to total protein and are given as a % change from the normoxia vehicle, presented as means with error bars representing SEM. *n*=6–9 from 3 experimental repeats. *, Significant difference compared with vehicle normoxia (vehicle N) (*p<0.05, **p<0.01, ***p<0.001, and ****p<0.0001). ^$^*p* < 0.05, ^$$^*p* < 0.01, ^$$$^*p* < 0.001, and ^$$$$^*p* < 0.0001) significant difference to vehicle OGD, one-way ANOVA with Dunnett's *post hoc* analysis. VEGF, vascular endothelial growth factor.

Protein levels from pericyte monocultures were not significantly altered by the OGD protocol or drug treatment (Supplementary Fig. S1A, D). A 4-h OGD increased levels of IL-6, VEGF, and IL-8 measured in cell culture medium 24-h post-OGD (Fig. 2A–F).

In pericyte monocultures, neither CBG nor CBDV (10 nM–3 μM) altered IL-6 levels post-OGD; however, both compounds increased IL-6 levels at 10 μM (*p*<0.0001; Fig. 2A, D). Pretreatment with CBG and CBDV (10 nM–10 μM) did not alter levels of VEGF (Fig. 2B, E).

At the lowest and highest concentrations tested, CBG pretreatment increased IL-8 levels compared with vehicle normoxia and vehicle OGD (*p*<0.05; Fig. 2C). At 100 and 300 nM, CBG did not alter increased levels of IL-8 produced by OGD (Fig. 2C). CBDV did not affect IL-8 levels post-OGD, although there was a trend to produce an increase in IL-8 at 10 μM (Fig. 2F).

### Astrocyte monocultures

**FIG. 3.**
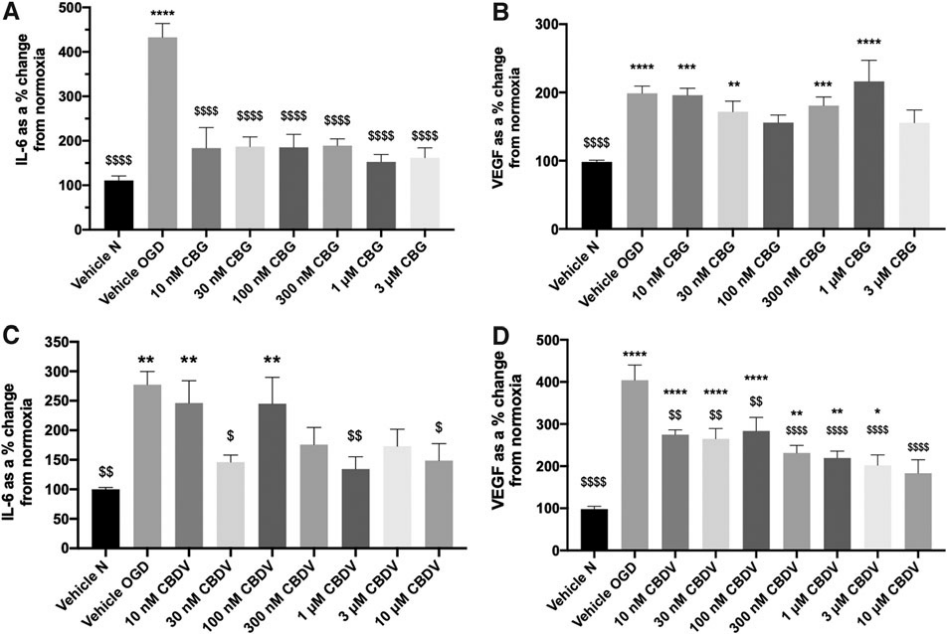
The effects of CBG and CBDV on astrocyte monocultures. **(A-D)** Medium 24 h after 8-h OGD were analyzed for IL-6 and VEGF. Data were normalized to total protein and are given as a % change from the normoxia vehicle, presented as means with error bars representing SEM. *n*=5–9 from 3 experimental repeats. *, Significant difference compared with vehicle normoxia (vehicle N) (*p<0.05, **p<0.01, ***p<0.001, and ****p<0.0001). ^$^*p* < 0.05, ^$$^*p* < 0.01, ^$$$^*p* < 0.001, and ^$$$$^*p* < 0.0001) significant difference to vehicle OGD, one-way ANOVA with Dunnett's *post hoc* analysis.

IL-6 levels were not statistically different to vehicle normoxia 24 h after 4-h OGD (70.03 pg·mL normoxia vs. 65.29 pg·mL OGD, data not shown), but levels of IL-6 were significantly increased 24 h after 8-h OGD (*p*<0.01; Figure 3A and C). Therefore, subsequent experiments in astrocytes were conducted using an 8-h OGD protocol.

An 8-h protocol significantly decreased protein levels in astrocyte cell lysates (*p*<0.01 versus vehicle normoxia; Supplementary Fig. S1B, E). Treatment with 10 μM CBG decreased protein content compared with both vehicle OGD and vehicle normoxia (*p*<0.0001; Supplementary Fig. S1B). Pretreatment with CBDV did not prevent the decrease in protein content caused by the 8-h OGD protocol (*p*<0.05 vs. vehicle normoxia); however, 30 nM, 1 and 10 μM CBDV did not exhibit a significant difference compared with vehicle normoxia (Supplementary Figure S1E).

Pretreatment with CBG 10 nM–3 μM attenuated astrocytic IL-6 levels (*p*>0.0001 vs. vehicle OGD; Fig. 3A); however, at 10 μM CBG significantly increased IL-6 (Supplementary Fig. S2A). CBDV reduced levels of IL-6 compared with vehicle OGD at 30 nM (*p*<0.05), 1 μM (*p*<0.01), and 10 μM (*p*<0.05; Fig. 3C). CBDV at 300 nM and 3 μM also appeared to decrease IL-6 levels, exhibiting no statistical difference to vehicle normoxia.

Astrocytic VEGF levels were significantly increased post-OGD (*p*<0.0001; Fig. 3B, D). CBG pretreatment appeared to attenuate VEGF levels at 100 nM and 3 μM, but this did not reach significance to vehicle OGD (Fig. 3B). Conversely, 10 μM CBG significantly increased VEGF compared with both vehicle normoxia and vehicle OGD (*p*<0.001; Supplementary Fig. S2B). Pretreatment with CBDV (10 nM–10 μM) decreased VEGF levels in a concentration-dependent manner. At 10 μM this was not significantly different to vehicle normoxia and significantly different to vehicle OGD (*p*<0.0001; Fig. 3D).

**FIG. 4.**
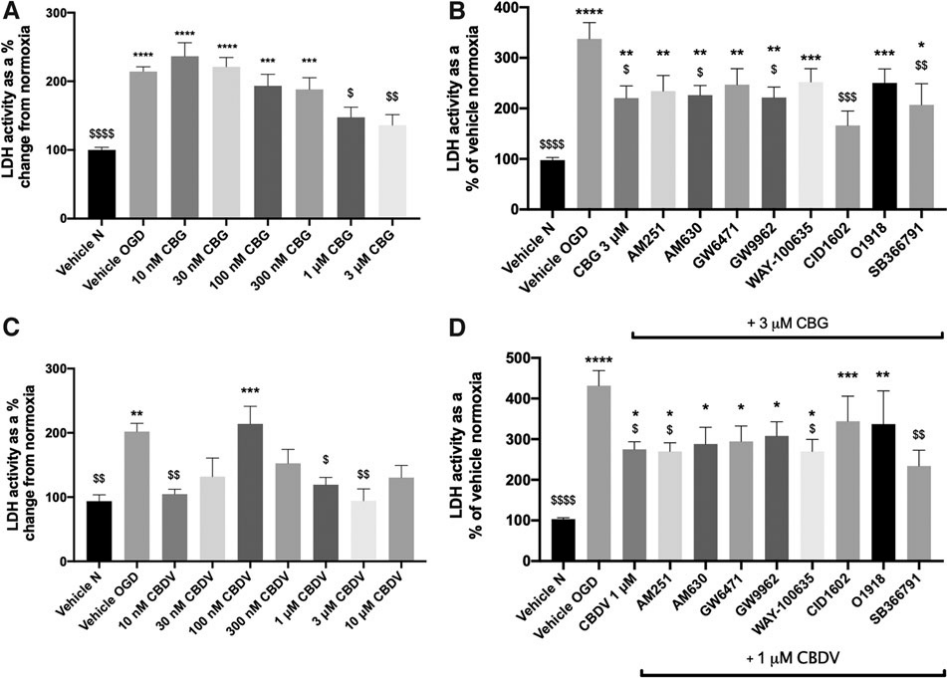
The effects of CBG and CBDV treatment alone **(A, B)** and with antagonists **(C, D)** on LDH release from astrocyte monocultures. Medium 24 h after 8-h OGD were analyzed LDH. Data were normalized to total protein and are given as a % change from the normoxia vehicle, presented as means with error bars representing SEM. *n*=5–6 from 3 experimental repeats. *, Significant difference compared with vehicle normoxia (vehicle N) (*p<0.05, **p<0.01, ***p<0.001, and ****p<0.0001). ^$^*p* < 0.05, ^$$^*p* < 0.01, ^$$$^*p* < 0.001, and ^$$$$^*p* < 0.0001) significant difference to vehicle OGD, one-way ANOVA with Dunnett's *post hoc* analysis. LDH, lactate dehydrogenase.

LDH was significantly elevated in astrocyte medium post-OGD (*p*<0.01; Fig. 4A, C). Pretreatment with 1 and 3 μM CBG significantly attenuated LDH activity (*p*<0.05; Fig. 4A); however, at 10 μM CBG significantly increased LDH activity (Supplementary Fig. S2C). CBDV exhibited a biphasic concentration response, decreasing LDH activity at lower (10 nM; *p*<0.01) and higher concentrations (*p*<0.05; 1 and 3 μM), but increasing levels at 100 nM (*p*<0.001; Fig. 4C).

None of the antagonists tested blocked CBG (3 μM)-mediated decreases in LDH; however, application of CID1602 (antagonist for GPR55) appeared to potentiate the effects of CBG (*p*<0.001; 3 μM CBG+CID1602 vs. vehicle OGD; Fig. 4B). In the presence of antagonists for GPR55, CID1602 and O1918, CBDV (1 μM)-mediated decreases in LDH were no longer significantly different to vehicle OGD (Fig. 4D). In addition, SB366791 appeared to potentiate the LDH-reducing effects of CBDV (*p*<0.01; 1 μM CBDV SB366791 vs. vehicle OGD, Fig. 4D).

**FIG. 5.**
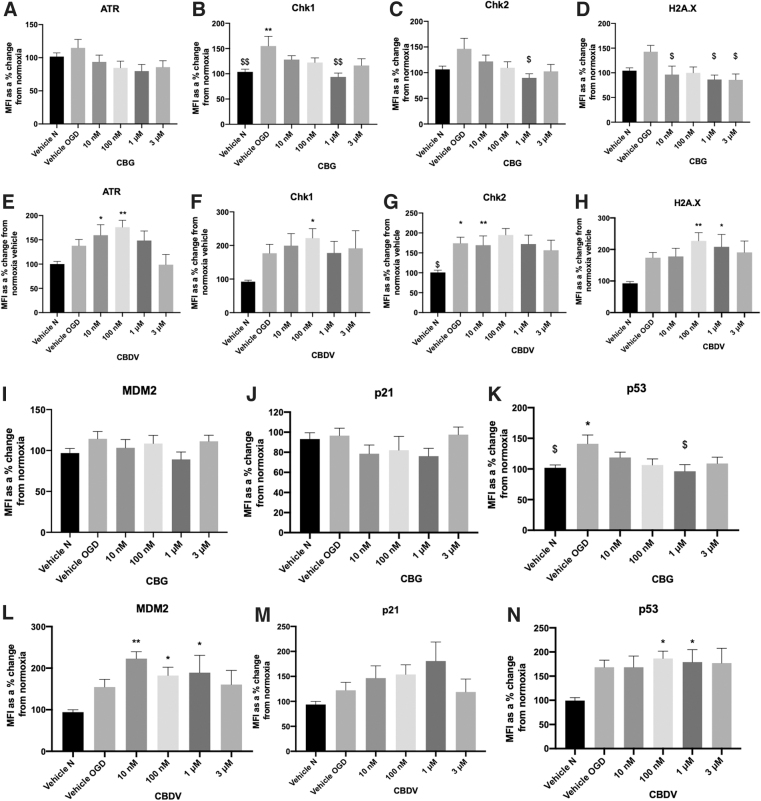
The effects of CBG and CBDV on DNA damage markers (ATR **[A,E]**, Chk1 **[B,F]**, Chk2 **[C,G]**, H2A.X **[D,H]**, MDM2 **[I,L]**, p21 **[J,M]**, and p53 **[K,N]** from astrocyte cell lysates, 24 h after 8-h OGD. Data were normalized to total protein and are given as MFI as a % change from the normoxia vehicle (vehicle N); means with error bars represent SEM. *n*=6–8 from 3 experimental repeats. *, Significant difference compared with vehicle normoxia (vehicle N) (*p<0.05, **p<0.01). ^$^*p*<0.05, ^$$^*p*<0.01 significant difference to vehicle OGD, one-way ANOVA with Dunnett's *post hoc* analysis. MFI, mean fluorescent intensity.

As CBDV and CBG reduced cell damage in astrocytes, we next investigated whether these compounds (at the most efficacious lower and higher concentrations tested) influenced levels of DNA damage proteins. Levels of DNA damage proteins, ATR, Chk1, Chk2, H2A.X, and p53 were increased in astrocyte cell lysates 24 h after 8-h OGD. MDM2 showed a trend for increasing post-OGD, but levels of p21 were not affected (Fig. 5A–N). Application of CBG (1 μM) before OGD significantly reduced levels of Chk1 and Chk2 compared with OGD vehicle (*p*>0.01, *p*<0.05; Fig. 5B, C). In addition, CBG pretreatment at 10 nM, 1 μM, and 3 μM decreased H2A.X levels that displayed trend for increasing post-OGD (*p*>0.05; Fig. 5D). Levels of p53 were also increased post-OGD (*p*>0.05) and attenuated by CBG in a concentration-dependent manner that was significant at 1 μM (*p*>0.05; Fig. 5K). By contrast, CBDV (10 nM, 100 nM) increased levels of ATR (*p* > 0.0001, *p* < 0.01; Fig. 5E) as well as increasing levels of Chk1 at 100 nM (*p* < 0.05) and Chk2 at 10 nM (*p* < 0.01; Fig.5F). CBDV (100 nM and 1 μM) also increased levels of H2A.X, p53 and MDM2 (*p* < 0.05; Fig. 5H, L, N).

## Discussion

In this study we assessed whether non-euphoric phytocannabinoids CBG and CBDV protected cells of the BBB in a cellular model of ischemic stroke. Despite promising preclinical data, drugs developed for one or more of the hallmarks of stroke have failed once they have reached clinical trials.^23,24^ Poor translational efficacy is likely to stem from the multifactorial pathophysiology of ischemic stroke and complicating factors among elderly patients, which are often overlooked in ischemic stroke modeling.^25^ These points emphasize the need to generate new, effective therapies for patients, which target multiple aspects of stroke pathogenesis.^26^

CBD has been widely studied as a neuroprotectant, partly because of its promiscuous pharmacology, tolerable safety profile in humans and absence of euphoric effects.^10,27,28^ However, other phytocannabinoids are beginning to gain significant interest as therapeutic agents. CBG has displayed prominent anti-inflammatory and antioxidant capabilities^20,29,30^ and the antiepileptic properties of CBDV have been well documented.^31–33^ Recently, CBDV has been shown to reduce inflammatory cytokine release in a model of intestinal inflammation.^34^ Our results demonstrate that CBDV and CBG exhibit protective properties against OGD-induced damage in astrocytes and HBMECs, modulating a range of biochemical parameters measured post-OGD. For CBDV, its cytoprotective effects appeared to partially involve GPR55, but a target for CBG was not identified. These data warrant their further investigation into these compounds as neuroprotectants and to assess their clinical applicability, specifically, their efficacy in *in vivo* models of ischemic stroke and whether they are protective when applied post-OGD.

Post-cerebral ischemia and elevated levels of proinflammatory cytokine IL-6 are associated with increased neuronal cell necrosis and are correlated with stroke severity, increases in mortality rate, poor performance, and functional disability.^35–38^ In this study, CBG and CBDV significantly decreased levels of IL-6 in astrocytes, suggesting that like CBD, CBDV and CBG may offer protection against inflammation caused by ischemic stroke.^12^ Increases in IL-6 post-ischemia have also been implicated in BBB breakdown and tight junction remodeling, including reduced expression of VE-cadherin, occludin, and claudin-5.^39^ Although there was a trend for CBDV and CBG to attenuate IL-6 levels in HBMECs, more pronounced reductions in IL-6 were observed in astrocytes. Astrocytes provide biochemical and mechanical support that help to maintain the BBB, as well as providing neurovascular crosstalk between neurons and cerebral blood vessels.^40^ Unlike in monoculture, *in vivo,* astrocyte endfeet are in direct contact with endothelial cells; thus, modulating the astrocyte inflammatory response *in situ* may act to preserve BBB integrity indirectly by soluble factors secreted by astrocytes or by preserving normal astrocyte function.

Mice lacking the receptor for adhesion molecule, MCP-1 (CCR2), have significantly reduced infarct sizes together with reduced BBB permeability and similarly, MCP-1 knockout mice have a reduced influx of hematogenic cells from systemic circulation and improved neurological outcome.^41,42^ Bonifačić et al. found a relationship between patients with poor outcomes 90 days after stroke and elevated levels of MCP-1 and a recent meta-analysis revealed that higher baseline circulating levels of MCP-1 correlated with a higher risk of ischemic stroke.^43,44^ Our data show that CBDV concentration dependently decreased levels of MCP-1 secreted by HBMECs when applied at the same time as initiating OGD, suggesting that CBDV might offer protection against MCP-1-related damage post-stroke and/or offer protection in individuals at a higher risk of ischemic stroke. These data are also consistent with that of a recent study showing that CBDV treatment attenuated MCP-1 mRNA levels in colonic tissue post-colitis.^34^ Of interest, this study also showed that CBDV was able to reduce intestinal permeability, an effect that may be replicated at the BBB, but this has yet to be investigated.

We also measured VEGF secreted by pericytes and astrocytes post-OGD reperfusion as elevations in VEGF are correlated with increased endothelial barrier permeability post-ischemia.^45–47^ Li and co-authors found that astrocyte-derived VEGF mediated endothelial barrier disruption, which was associated with decreases in occludin and claudin-5.^47^ Interestingly, whilst CBG and CBDV did not affect pericyte-derived VEGF, CBDV decreased VEGF secretion in astrocytes in a concentration-dependent manner and CBG exhibited a trend for decreasing VEGF at 100 nM and 3 μM. As VEGF is known to facilitate BBB opening these compounds may offer protection against BBB breakdown post-ischemia; however, the mechanisms in which these compounds decrease VEGF remains to be elucidated.

During IR injury cells undergo a combination of apoptosis and necrosis, causing various cellular components to be released into the extracellular space. One of these components, LDH, is often used as a marker of cell damage. Previous studies have shown that IR models cause LDH leakage into cell culture medium^48,49^ and clinically, LDH has been trialled as a marker of ischemic severity.^50,51^ Pretreatment with CBDV and CBG offset increases in LDH, suggesting both compounds mitigate cellular damage produced by OGD reperfusion. Application of receptor antagonists revealed that CBDV appeared to mediate its effects on LDH levels by GPR55; however, none of the antagonists tested blocked the effect of CBG. This could be explained by the nonspecific antioxidant properties of cannabinoids, namely owing to their phenolic rings and hydroxyl moieties.^17,52^ Indeed, previous studies have shown that CBD increases antioxidant enzymes in BV2 microglial cells,^53^ as well as attenuating oxidative stress and increasing mitochondrial bioenergetics in OGD reperfusion-damaged neurons.^18^ Similarly, CBD, CBDV, and CBG were able to prevent oxytosis in a preclinical drug screen for Alzheimer's disease and CBG exhibited antioxidant capacity in neuroblastoma cells.^54,55^ More data are clearly needed on the specific and nonspecific mechanisms in which these compounds mediate their protective effects, particularly whether their antioxidant status is responsible for reducing cell damage in the context of ischemia.

Ischemia is a pathophysiological stressor and as a consequence, nonspecific single- and double-strand DNA breaks (ssDNA/dsDNA breaks) and replication-associated DNA damage responses (DDRs) occur. DNA damage can activate the DDR pathway and DDR response proteins ATR, Chk1, Chk2, H2A.X, MDM2, p21 and p53 that govern elements of DNA repair, cell cycle arrest, apoptotic and necrotic cell death.^56–59^ These processes are central in IR injury and early studies found that neurons are the first to exhibit signs of DNA damage (0.5–8 h reperfusion) followed by astrocytes (24 h reperfusion).^60^ Thus, we next investigated the effect of CBDV and CBG on DDR proteins post-OGD in astrocytes.

In support of previous studies, our OGD protocol (and subsequent reperfusion period) increased levels of almost all measured DDR proteins in astrocyte monoculture lysates.^61^ In stroke patients, Huttner and colleagues found evidence of ATM/ATR activity in the penumbra of cortical neurons 7–10 days post-ischemia.^62^ Studies have also shown p53 activation is implicated in ischemia-induced neuronal cell death, with elevated levels of p53 also present in reactive astrocytes and microglia.^63,64^ Ahn and colleagues found that inhibition of p53 by pifithrin-α reduced OGD-induced cell death in cultured astrocytes, and as a secondary effect reduced elevated levels of glutamate and glial fibrillary acidic protein (GFAP), which were also increased post-OGD.^65^ To our knowledge, this is the first study to show that CBG pretreatment reduced levels of Chk1, Chk2, H2A.X, and p53 in astrocytes post-OGD. It is likely that these decreases in DNA damage proteins were caused indirectly, possibly because of the overall reductions in cellular damage and inflammation, as well as the known antioxidant properties of CBG that have both been demonstrated in other studies.^20,66^ Nevertheless, direct modulation of these proteins should not be ruled out particularly as PPAR-γ, a known target for phytocannabinoids, has been implicated in ATM signaling and the DDR.^67^

Pretreatment with CBDV significantly increased expression of the majority of DNA damage proteins in astrocytes and exhibited a trend for increasing p21. CBD was recently found to increase protein expression of ATM and p21, but not p53 in an *in vitro* model of gastric cancer, suggesting CBD promotes cell cycle arrest at the G_0–_G_1_ phase.^68^ Our data suggest that CBDV acts in a similar manner; however, it is important to emphasize that p21 has roles in both enhancing and inhibiting apoptosis depending on the type of stressor; thus, generating this response in a cancer cell model will be different to responses of astrocytes subjected to OGD. Low dose *N*-methyl-d-aspartate (NMDA) to simulate ischemic preconditioning was shown to increase MDM2 protein expression, preventing p53 stabilization in mouse cortical neurons and ischemia-induced apoptotic cell death.^69^ CBDV significantly increased levels of MDM2, which is a key protein involved in p53 degradation and thus promotion of cell survival. Future studies should clarify the implications of CBDVs ability to increase levels of DNA damage proteins in ischemia and establish whether modulating DNA damage and repair in astrocytes can influence post-stroke injury and recovery.

## Conclusions

This study provides novel data on the neuroprotective and anti-inflammatory properties of CBG and CBDV in an *in vitro* model of IR. These data, together with evidence from other studies, corroborate the protective properties of these compounds and further studies are needed to elucidate the mechanism of action of CBG and CBDV and whether they can modulate BBB permeability in more clinically relevant *in vivo* models of ischemic stroke. There is lack of effective treatments for ischemic stroke, a condition that will increase in prevalence in coming years, to which cannabinoids may offer a unique therapeutic strategy.

## Supplementary Material

Supplemental data

Supplemental data

## Abbreviations Used

Δ^9^-THC: delta^9^-tetrahydrocannabinol
ANOVA: analysis of variance
BBB: blood–brain barrier
BCA: bicinchoninic acid
CBDV: cannabidivarin
CBG: cannabigerol
CNS: central nervous system
DDR: DNA damage response
ELISA: enzyme-linked immunosorbent assay
GFAP: glial fibrillary acidic protein
HA: human astrocytes
HBMECs: human brain microvascular endothelial cells
ICAM-1: intracellular adhesion molecule-1
IR: ischaemia–reperfusion
LDH: lactate dehydrogenase
MCP-1: monocyte chemoattractant protein-1
MFI: mean fluorescent intensity
NMDA: *N*-methyl-d-aspartate
OGD: oxygen-glucose deprivation
TEER: trans epithelial resistance
VEGF: vascular endothelial growth factor
VCAM-1: vascular adhesion molecule
