# Supplementary figures and images for: Protective Effects of Cannabidivarin and Cannabigerol on Cells of the Blood–Brain Barrier Under Ischemic Conditions
Source: Cannabis Cannabinoid Res. 2021 Aug 5;6(4):315–26. doi: 10.1089/can.2020.0159 (PMC8380798; doi:10.1089/can.2020.0159)

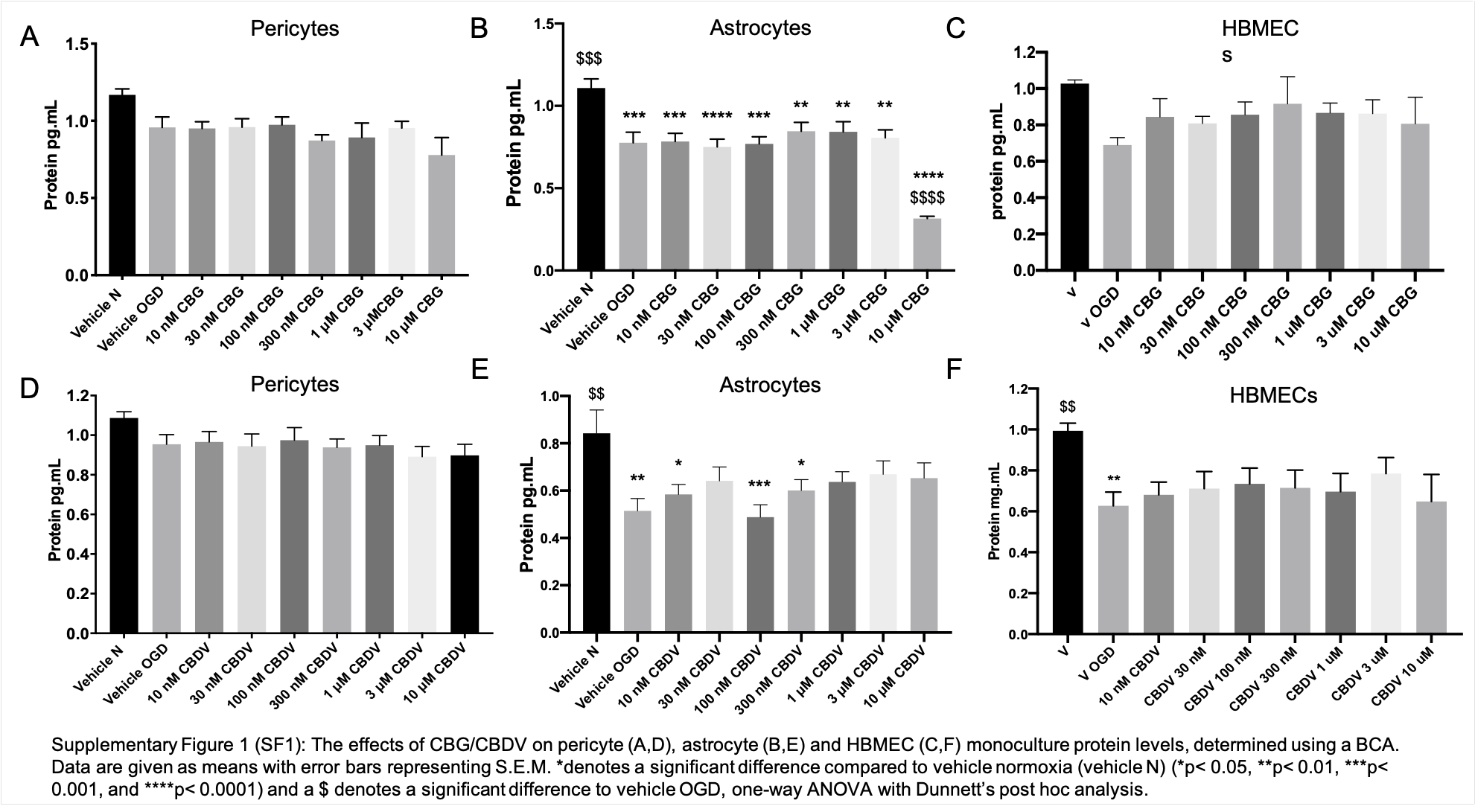

Supplement: Supplemental data [file Supp_Fig1.tiff]

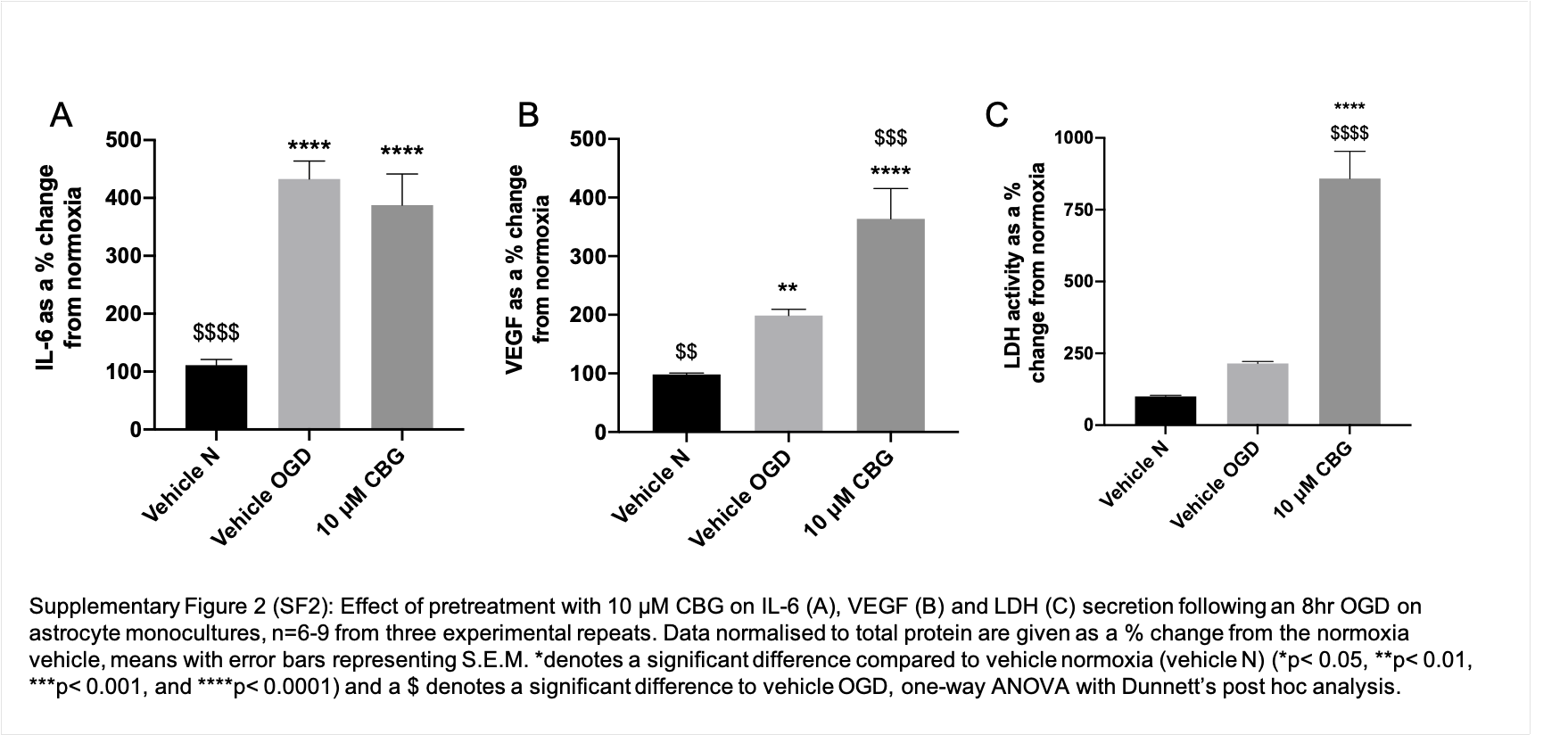

Supplement: Supplemental data [file Supp_Fig2.tiff]
